# Association of inflammatory biomarkers and disease activity with subclinical myocardial dysfunction in psoriatic arthritis

**DOI:** 10.1038/s41598-023-37412-6

**Published:** 2023-06-26

**Authors:** Ivan Pletikosic, Daniela Marasovic Krstulovic, Darija Bakovic, Zora Susilovic Grabovac, Leida Tandara, Dusanka Martinovic Kaliterna

**Affiliations:** 1 Cardiovascular Diseases Department, University Hospital Split, Split, Croatia; 2 Department of Internal Medicine, University Hospital Split, Split, Croatia; 3 Medical Laboratory Diagnostic Division, University Hospital of Split, Split, Croatia; 4 Department of Physiology, School of Medicine, University of Split, Split, Croatia; 5 School of Medicine, University of Split, Split, Croatia

**Keywords:** Cardiology, Cardiovascular biology

## Abstract

We examined the role of adipokines and pro-inflammatory cytokines in psoriatic arthritis-associated subclinical myocardial dysfunction, and the relationship between these variables and psoriatic arthritis (PsA) disease activity. Fifty-five PsA patients without cardiovascular risk factors and 25 controls underwent standard and speckle tracking echocardiography with global longitudinal strain (GLS) calculated. Standard anthropometric data and Disease Activity in Psoriatic arthritis (DAPSA) scores were recorded, with low disease activity defined as DAPSA ≤ 14 and moderate and high disease activity DAPSA > 14. Standard biochemical tests, adiponectin, resistin, leptin, tumor necrosis factor (TNF) alfa, interleukin 17 A (IL-17A), B lymphocyte chemoattractant (BLC), and monokine induced by intereferon gamma (MIG) were analyzed. Median age was 53.0 (46.0–61.0), median PsA duration 6.0 (4.0–13.0) years and median DAPSA score 25.5 (13.0–41.5). Lower GLS, tricuspid annular plane systolic excursion (TAPSE) and left ventricular ejection fraction (LVEF) were found in moderate and high PsA disease activity compared to low PsA disease activity and controls. PsA patients with GLS < 20 had higher body mass index (BMI), DAPSA score and uric acid levels, and lower adiponectin levels. Although patients with GLS < 20 had higher IL-17A levels, it was not statistically significant (*P* = 0.056). However, when we included healthy controls and analyzed differences based on a GLS cut-off of 20% in the entire population, the difference in IL-17A became statistically significant, 0.17 pg/mL (0.06–0.32) vs. 0.43 pg/mL (0.23–0.65), *P* = 0.017. The association between DAPSA score and GLS and IL-17 remained significant in multivariate analysis. Moreover, the association between GLS and IL-17 and adiponectin was significant after adjustment for age and BMI. Patients with moderate and high PsA disease activity have reduced myocardial function, lower adiponectin, and higher IL-17A levels.

Psoriatic arthritis (PsA) is a chronic inflammatory arthritis associated with psoriasis. The prevalence of PsA in the general population ranges from 0.16 to 0.32%^1^. Patients with PsA have a 43% higher risk of cardiovascular disease (CVD) compared to the general population^2^, and PsA is an independent risk factor for major cardiovascular (CV) events including myocardial infarction and stroke^3,4^. Furthermore, increased morbidity and mortality has been reported, mainly attributed to CVD^4^. Moreover, patients with PsA without traditional CV risk factors or clinically evident CVD exhibit endothelial dysfunction and have increased carotid artery intima-media thickness compared to healthy controls^5^. Chronic inflammation plays a pivotal role in the pathogenesis of atherosclerosis in PsA, acting independently and/or synergistically with traditional risk factors^6^, and evidence supports a link between the extent of inflammation and CV risk^7^. Studies have shown that patients with psoriasis and PsA, with elevated inflammatory biomarkers, have high atherosclerosis disease burden^7,8^.

Although the pathogenesis of atherosclerosis in PsA has been well studied, little is known about the impact of PsA on cardiac function. Several studies have demonstrated a high prevalence of subclinical left ventricular dysfunction in patients with PsA without clinical evidence of CV disease^9–13^. Two-dimensional speckle tracking echocardiography (STE) is a novel method for detecting ventricular dysfunction by echocardiographic assessment of myocardial deformation (strain)^11^. The use of STE to detect subclinical myocardial dysfunction in patients with rheumatic disease has been proposed to improve patient stratification and risk management^14^.

Several studies have linked inflammatory disease burden and PsA disease activity with subclinical myocardial dysfunction^11,13,15^, but it is still unclear which mechanism are involved in this process. Obesity has been found to predict worse clinical outcomes and treatment responses in patients with psoriasis and PsA^16^, possibly due to the role of adipokines. Adipose tissue is metabolically active and an important source of proinflammatory and anti-inflammatory adipokines involved in the inflammation associated with autoimmune and cardiovascular disease^17,18^. Pro-inflammatory cytokines, specifically interleukin 17A (IL-17A), contributes to the pathogenesis of various inflammatory diseases including PsA, and its effects on vascular and cardiac cells may contribute to the increased cardiovascular risk seen in these patients^16^. Although studies have found elevations of some adipocytokines and inflammatory markers in patients with PsA^17,19–21^, their association with disease activity and myocardial impairment is unknown. Therefore, we aimed to analyze the association of adipokines and other proinflammatory cytokines with subclinical myocardial dysfunction in patients with low and moderate and high PsA disease activity compared to healthy controls.

## Materials and methods

### Study population

**Figure 1:**
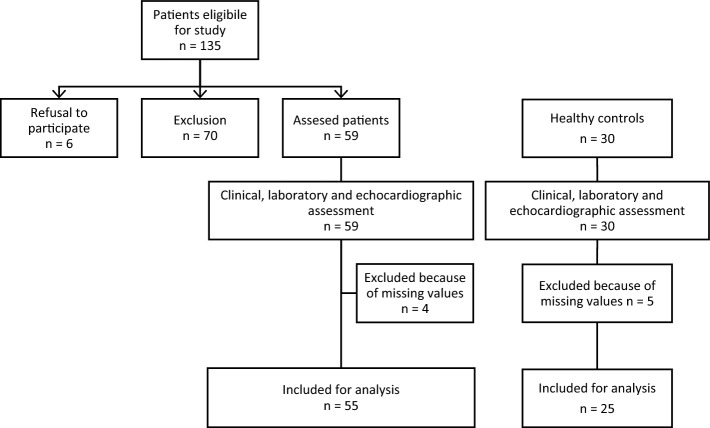
Flowchart of patients in the present study.

Between November 2017 and April 2020, 160 Caucasian patients ages 18 to 65 years were diagnosed with PsA and satisfied CASPAR classification^22^. Exclusion criteria was the presence of arterial hypertension, coronary heart disease, valvular heart disease, chronic heart failure, history of transient ischemic attack (TIA) or cerebrovascular insult (CVI), diabetes mellitus, moderate or severe chronic kidney disease defined as estimated glomerular filtration rate (eGFR) < 60 ml/min, aortic/peripheral arterial disease, clinically significant arrhythmia, smoking or alcohol use (≥ 3 drinks per week). After applying exclusion criteria, only 55 subjects with PsA were considered eligible for this study. Twenty-five healthy Caucasian controls, ages 18–65 years, without cardiovascular disease were randomly selected. The flow diagram of patient inclusion in the study is depicted in Fig. 1. Ethical approval was obtained from the University of Split School of Medicine Ethics Committee and was conducted in compliance with the Declaration of Helsinki (2000) of the World Medical Association. All participants provided written informed consent.

### Clinical assessment

Disease activity was assessed at baseline using Disease Activity Index in Psoriatic Arthritis (DAPSA). The DAPSA score is calculated using tender joint count (TJC68) and swollen joint count (SJC66), patient's global and pain scores on a visual analogue scale (VAS), and the C‐reactive protein (CRP) level^23^. Patients were divided into two groups based on disease activity: DAPSA ≤ 14 for low disease activity and remission, and DAPSA > 14 for moderate and high disease activity. Signs and symptoms of psoriasis (skin abnormality and nail lesions) were examined and the Psoriasis Area and Severity Index (PASI) was calculated to evaluate the severity of psoriasis^24^. Weight, height, blood pressure, age, sex, body mass index (BMI), drug history and disease duration were recorded for PsA patients. Age, sex, blood pressure and BMI were recorded for healthy controls.

### Laboratory

#### Blood sampling

Blood samples were collected between 7 and 10 am after a 12-h fast. Serum samples were centrifuged at 1800 g. Routine laboratory tests were performed the same day and serum aliquots were separated from cells and stored at −80 °C for further analysis.

#### Biochemical and haematological analysis

Routine laboratory parameters were determined using standard laboratory methods on the biochemical analyzer AU680 (Beckmann Coulter, Brea, CA, USA). CRP levels were measured turbidimetrically on the biochemical analyzer AU680 (Beckmann Coulter, Brea, USA). The complete blood count was obtained by the hematology analyzer ADVIA 2120i (Siemens Healthcare, Erlangen, Germany).

Commercially available enzyme-linked immunosorbent assay kits were used to determine IL-17A (Quantikine HS ELISA, R&D Systems, Minneapolis, MN, USA), tumor necrosis factor alfa (Quantikine ELISA, R&D Systems, Minneapolis, MN, USA), resistin (Quantikine ELISA, Human Resistin Immunoassay, R&D Systems, Minneapolis, MN, USA), leptin (Quantikine ELISA, Human Leptin Immunoassay, R&D Systems, Minneapolis, MN, USA), and adiponectin levels (Quantikine ELISA, Human Total Adiponectin/Acrp30 Immunoassay, R&D Systems, MN, Minneapolis, USA). B lymphocyte chemoattractant (BLC) and monokine induced by gamma interferon (MIG) levels were measured using the addressable laser bead immunoassay on the Luminex analyser (ProcartaPlex Multiplex Immunoassay, eBioscience, San Diego, CA, USA).

#### Echocardiography

Transthoracic echocardiography was performed using Vivid 9 (GE Medical System, Milwaukee, USA). Patient data was digitally stored and analyzed using Echo Pac workstation (GE Medical Systems, EchoPac PC, version 112). Echocardiographic assessments included:left atrial (LA) and left ventricular internal diameter at end-diastole (LVIDd), interventricular septum thickness at end-diastole (IVSd), left ventricular (LV) ejection fraction (LVEF), tricuspid annular plane systolic excursion (TAPSE) measurement in M-mod, color Doppler imaging of all valves and transmitral flow (E/A) and tissue Doppler (E/e ratio) for diastolic dysfunction assessment. All measurements were assessed according to the recommendations of the European association of cardiovascular imaging^15^. Subclinical left ventricular dysfunction was assessed using STE and global longitudinal strain (GLS). STE was measured using a commercially available speckle tracking system in an ECHOPAC workstation. Images were captured from three projections, four chamber, two chamber and the apical long axis at a rate of 50–60 frames per second. The peak systolic longitudinal strain for each segment was displayed based on a 17-segment model for each plane, and the results of all 3 planes were combined in a single bull’s-eye summary. Global longitudinal peak strain was automatically calculated as an average value of peak longitudinal strain in all 3-image planes (apical 2-, 4- chamber and long axis views). Typically, strain values are described by negative values, where the more negative values depict better LV performance. Based on previously published data using this software system, a GLS value of −19.7% was considered normal^25^. We changed negative into positive GLS values in order to improve the technical and graphical presentation of the manuscript. All echocardiographic studies and measurements were performed by an experienced cardiologist who was blinded to previously obtained data.

#### Statistical analysis

Patient characteristics were assessed using descriptive statistics presented as median and interquartile range (25–75%, IQR). Independent continuous variables were compared with Kruskal–Wallis test and Mann–Whitney test, when appropriate. Categorical variables were compared with Fisher exact test. Patients were categorized into two groups based on disease activity using DAPSA score cut-offs proposed in previous studies. Receiver operating characteristic (ROC) analysis was performed to determine the optimal cut-off value for GLS, which had the best ability to demonstrate differences in laboratory and clinical parameters. The association between disease activity, GLS and interleukins was assessed further with univariate and multivariate linear analysis. Logarithmic transformation has been performed with natural logarithm for all variables in order to transform data in a fashion that follows normal distribution. Multivariate analysis was carried out by using backwards stepwise linear regression on logarithmically transformed data. We used two different approaches for multivariate analysis. First multivariate model has been created with independent variables that showed significant association in univariate linear regression analysis. Although we have performed a logarithmic transformation of the date in order to artificially create normal distribution in order to make linear regression analysis possible, univariate and multivariate linear regression analysis can be misleading in case of relatively small sample size and in case of excess of independent variables in the model. Hence, we have performed additional two different multivariate models which comprised of parameters that showed statistical significance after Kruskal–Wallis and Mann–Whitney tests. Final conclusions were made based on the last two models of multivariate analysis. *P* value of < 0.05 was considered statistically significant. The statistical analysis was done using SPSS, Version 20 (IBM Corporation, Armonk, NY, USA).

## Results

Our study included 33 (60%) females and 22 (40%) males with PsA. Median age was 53.0 (46.0–61.0), median PsA duration 6.0 (4.0–13.0) years and median DAPSA score 25.5 (13.0–41.5). Fifty (89.4%) patients were receiving disease-modifying antirheumatic drugs (DMARD), nine (16.4%) systemic glucocorticoids, and 23 (41.8%) biologic therapy.

First, we investigated the associations between age, sex, and BMI with other parameters in patients with PsA. Age, sex and BMI were not associated with the duration nor activity of psoriasis and PsA. Male sex was associated with higher uric acid and lower high-density lipoprotein (HDL) levels. When assessing echo parameters, age was negatively associated with mitral valve (MV) E/A ratio (ρ = −0.425, *P* = 0.002), and male sex was associated with greater LVIDd and LA diameter. BMI also positively correlated with LA diameter (ρ = 0.546, *P* < 0.001). Male sex was associated with lower adiponectin and leptin levels, and higher BMI with lower adiponectin levels (−0.459, *P* = 0.002). There were no significant correlations between age, sex, and BMI and inflammatory biomarkers.

### Differences based on disease activity

The study population was divided into two cohorts based on disease activity: DAPSA ≤ 14 for low disease activity and remission, and DAPSA > 14 for moderate and high disease activity. Patients with low disease activity had significantly lower BMI and CRP levels, a lower prevalence of dactylitis, and shorter disease duration. There were no significant differences in treatment protocols and other laboratory parameters (Table 1).

**Table 1:** Characteristics of the studied population divided based on disease activity.

	Low (DAPSA ≤ 14) (N = 18)		Moderate and high (DAPSA > 14) (N = 37)		*P*
Age (years), median (IQR)	50.5	47.0–63.0	54.0	46.0–59.0	0.699
Female sex N (%)	12	66.7	21	56.8	0.565
Body mass index (BMI), median (IQR)	23.4	22.1–24.3	25.7	23.9–27.7	0.007*
Systolic BP (mmHg), median (IQR)	130	110–135	130	120–140	0.265
Diastolic BP (mmHg), median (IQR)	70	70–80	70	70–80	0.957
Duration of PsA (years), median (IQR)	3.5	2.3–7.0	7.0	5.0–13.0	0.013*
Duration of psoriasis (years), median (IQR)	4.5	3.0–18.5	12.0	6.0–20.5	0.106
PsA onset in relation to psoriasis diagnosis					0.237
Prior N (%)	5	27.8	6	16.2	
During N (%)	5	27.8	8	21.6	
After N (%)	6	33.3	23	62.2	
Dactylitis present N (%)	1	5.5	18	48.6	0.004*
Sacroiliitis present N (%)	4	22.2	14	37.9	0.529
Systemic glucocorticoids N (%)	2	11.1	7	18.9	0.702
Disease Modifying Anti-Rheumatic Drugs (DMARD)					0.425
None N (%)	3	16.7	2	5.4	
Leflunomid N (%)	2	11.1	8	21.7	
Methotrexate N (%)	6	33.3	15	40.5	
Salazopyrin N (%)	3	16.7	4	10.9	
Methotrexate + leflunomide N (%)	0	0.0	2	5.4	
Salazopyrin + leflunomid N (%)	0	0.0	2	5.4	
Biologic therapy N (%)	6	33.3	17	45.9	1.000
Duration of biologic therapy (years), median (IQR)	3.0	1.0–6.0	3.0	2.0–7.5	0.755
Type of biologic therapy					0.216
Certolizumab N (%)	0	0.0	2	5.4	
Sekukinumab N (%)	1	5.5	4	10.8	
Etanercept N (%)	1	5.5	1	2.7	
Adalimumab N (%)	0	0.0	7	18.9	
Infliksimab N (%)	2	11.1	1	2.7	
Golimumab N (%)	2	11.1	1	2.7	
Routine laboratory evaluation					
Erythrocyte sedimentation rate, median (IQR)	9.5	5.0–13.9	11.0	4.0–22.0	0.382
Thrombocytes (×10^9^/L), median (IQR)	232.0	213–283	243.0	210–270	0.920
Fasting glucose (mmol/L),	5.1	4.6–5.5	5.3	4.7–5.9	0.579
Uric acid (µmol/l),	239.0	220–320	289.5	251–366	0.058
C-reactive protein (CRP) (mg/L), median (IQR)	1.4	0.7–2.5	4.5	2.5–10.8	< 0.001*
Total cholesterol (mmol/L), median (IQR)	6.0	4.9–6.2	5.6	4.7–6.6	0.794
Triglycerides (mmol/L), median (IQR)	1.0	0.8–1.3	1.3	0.9–2.3	0.056
High-density lipoprotein (HDL) (mmol/L), median (IQR)	1.5	1.2–1.7	1.3	1.1–1.7	0.241
Psoriasis area and severity index (PASI score), median (IQR)	0.3	00–1.25	1.3	0.6–4.8	0.009

* statistically significant (P < 0.05).

**Figure 2:**
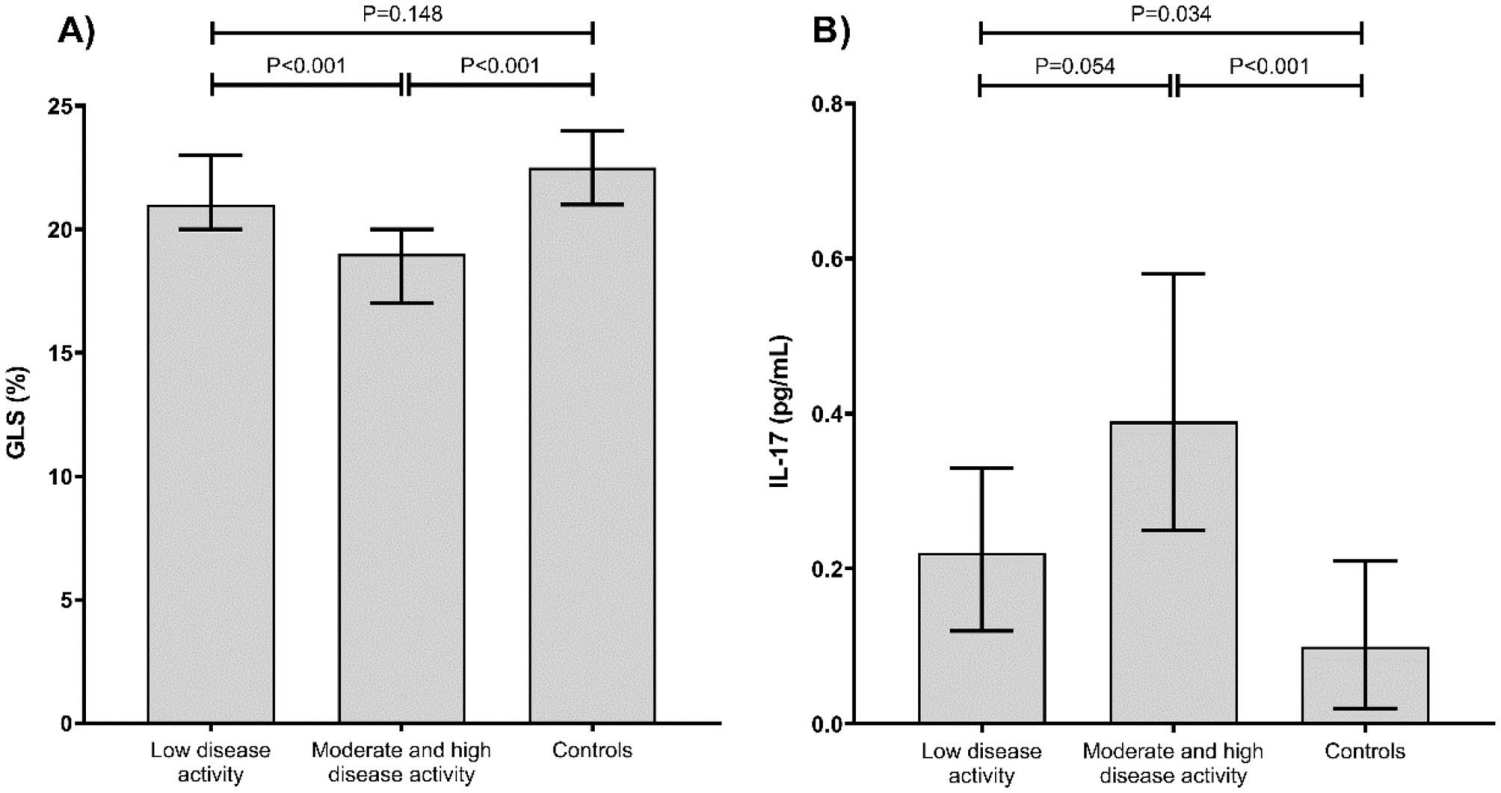
Relationship between global longitudinal strain (GLS) (**a**) and IL-17 levels (**b**) in patients with low, moderate and high disease activity, and controls.

When assessing echocardiographic parameters, interleukin and adipokine levels, we found that patients with moderate and high disease activity had lower GLS when compared to patients with low disease activity and controls (Table 2, Fig. 2a). Moreover, patients with moderate and high disease activity had lower TAPSE and LVEF when compared to patients with low disease activity and controls (Table 2). Serum BLC levels were lower in patients with low disease activity when compared to controls, but there were no differences in BLC levels in patients with low and moderate and high disease activity, or between patients with moderate and high disease activity and controls (Table 2). Serum IL-17A was higher in patients with both low and moderate to high disease activity in comparison to controls (Table 2, Fig. 2b), but the difference between disease activity did not reach statistical significance (*P* = 0.054). We found no statistically significant differences in other inflammatory parameters and adipokines (Table 2).

**Table 2:** Comparison of echocardiographic parameters, inflammatory parameters and adipokine levels in patients and controls.

	Low disease activity (A)		Moderate and high disease activity (B)		Controls (C)		*P* A vs. C	*P* A vs. B	*P* B vs. C
Age (years), median (IQR)	50.5	47.0–63.0	54.0	46.0–59.0	40.5	33.0–53.0	0.015	0.699	< 0.001*
BMI (kg/m^2^), median (IQR)	23.4	22.1–24.3	25.7	23.9–27.7	24.2	22.5–26.2	0.314	0.007	0.092
Systolic BP (mmHg), median (IQR)	130	110–135	130	120–140	125	120–130	0.610	0.265	0.041
Diastolic BP (mmHg), median (IQR)	70	70–80	70	70–80	70	65–75	0.227	0.957	0.084
LVIDd (mm), median (IQR)	4.9	4.5–5.1	5.0	4.6–5.4	4.6	4.4–4.9	0.306	0.374	0.037
LA Diam (mm),median (IQR)	3.7	3.1–4.1	3.7	3.4–4.0	3.2	3.0–3.4	0.047	0.511	< 0.001*
TAPSE (cm), median (IQR)	2.7	2.6–2.6	2.5	2.2–2.7	3.2	2.9–3.4	< 0.001*	0.014	< 0.001*
MV E/A Ratio, median (IQR)	1.2	1.0–1.4	1.2	1.0–1.4	1.4	1.2–1.9	0.055	0.510	0.002
E/E Avg, median (IQR)	6.8	5.9–8.6	7.0	6.0–7.8	6.8	5.8–7.9	0.534	0.850	0.737
GLS (%), median (IQR)	21.0	20.0–23.0	19.0	17.0–20.0	22.5	21.0–24.0	0.148	< 0.001*	< 0.001*
LVEF_BiP (%), median (IQR)	66.5	59.0–69.0	58.0	53.0–64.0	66.0	64.0–69.0	0.666	0.021	< 0.001*
BLC (pg/ml), median (IQR)	73.3	56.5–81.1	88.8	53.8–139.6	96.5	70.2–140.2	0.040	0.121	0.732
MIG (pg/ml), median (IQR)	6.7	4.0–18.1	7.3	3.6–11.6	3.6	1.6–9.8	0.082	0.841	0.076
IL-17 (pg/ml), median (IQR)	0.22	0.12–0.33	0.39	0.25–0.58	0.10	0.02–0.21	0.034	0.054	< 0.001*
TNF L (pg/ml), median (IQR)	22.8	18.1–73.5	21.4	13.5–31.6	18.6	12.5–27.8	0.081	0.229	0.579
Adiponectin (ng/L), median (IQR)	11.7	5.6–13.5	6.4	4.8–8.7	7.0	5.2–10.9	0.099	0.153	0.696
Leptin (pg/L), median (IQR)	10.7	9.1–15.2	14.1	8.6–24.1	12.2	5.9–21.0	0.691	0.565	0.369
Resistin (pg/ml), median (IQR)	10.0	8.4–10.6	10.0	7.1–11.9	11.2	10.0–13.8	0.100	0.812	0.127

*BMI* body mass index, *BP* blood pressure, *LVIDd* left ventricular internal diameter at end-diastole, *LA* left atrium, *TAPSE* tricuspid annular plane systolic excursion, *MV* mitral valve, *E/E* early diastolic mitral inflow velocity to early diastolic mitral annulus velocity, *GLS* global longitudinal strain, *LVEF BiP* left ventricular ejection fraction biplane, *BLC* B lymphocyte chemoattractant, *MIG* monokine induced by intereferon gamma, *IL* interleukin, *TNF L* tumor necrosis factor alfa, *IQR* interquartile range.

* statistically significant (P < 0.05).

### Association of adipokines and cytokines with cardiac function

**Figure 3:**
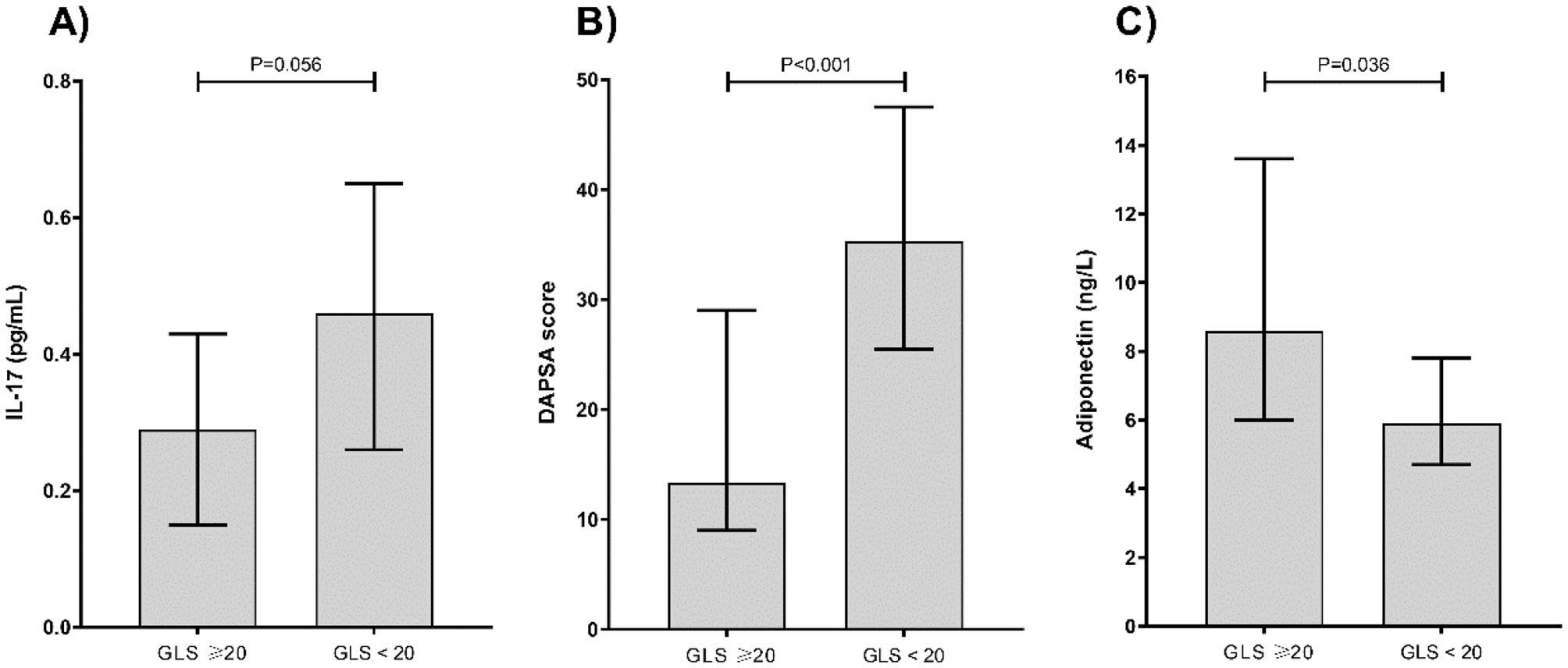
Relationship between IL-17 (**a**), DAPSA score (**b**), and adiponectin (**c**) with global longitudinal strain (GLS) in a subgroup analysis using a GLS cut-off of 20.0.

GLS was the best echocardiographic parameter to distinguish patients with low and moderate to high disease activity. A ROC analysis disclosed that a GLS cut-off of 20.0% had the best diagnostic accuracy to differentiate low and moderate to high disease activity (sensitivity 78.4%, specificity 72.2%). Hence, we performed a subgroup analysis in patients with PsA and GLS ≥ 20% and GLS < 20%. PsA patients with GLS < 20% had greater BMI, DAPSA score and serum uric acid levels, and lower adiponectin levels (Table 3). Although patients with GLS < 20% had higher IL-17A levels, it did not reach statistical significance (0.056). However, when we included healthy controls and analyzed differences based on a GLS cut-off of 20% in the entire study population, the difference in serum IL-17A levels became statistically significant, 0.17 pg/mL (0.06–0.32) vs. 0.43 pg/mL (0.23–0.65), P = 0.017. The association between IL-17A, adiponectin and DAPSE score in the subgroup analysis using a GLS cut-off of 20.0% is depicted in Fig. 3.

**Table 3:** Comparison of PsA patients with GLS ≥ 20 and GLS < 20.

	GLS ≥ 20 N = 29		GLS < 20 N = 26		*P*
Age (years), median (IQR)	52.0	47.0–61.0	53.5	43.0–60.0	0.987
Female sex	20	69.0%	13	50%	0.152
BMI, median (IQR)	23.5	22.2–25.1	26.3	25.3–27.7	0.001*
Duration of PsA (years), median (IQR)	7.0	3.0–12.0	6.0	4.0–13.0	0.872
Duration of psoriasis (years), median (IQR)	9.5	3.0–20.0	11.5	6.0–20.0	0.336
PsA onset in relation to psoriasis diagnosis					0.068
Prior N (%)	9	31.0%	2	7.7%	
During N (%)	6	20.7%	7	26.9%	
After N (%)	12	41.3%	17	65.4%	
Systolic BP (mmHg), median (IQR)	130.0	120.0–135.0	130.0	120.0–140.0	0.904
Diastolic BP (mmHg), median (IQR)	70.0	70.0–80.0	70.0	70.0–75.0	0.311
PASI score, median (IQR)	.6	0.0–2.5	1.3	0.6–5.5	0.078
DAPSA score, median (IQR)	13.3	9.0–29.0	35.3	25.5–47.5	< 0.001*
Disease activity					
Low	17	58.6%	1	3.8%	< 0.001*
moderate and high	12	41.4%	25	96.2%	
Glucocorticoids	9	31.0%	9	34.6%	0.360
Biologics	10	34.4%	13	50.0%	0.678
Duration of biologic therapy (years), median (IQR)	0.0	0.0–2.0	1.0	0.0–3.0	0.338
ESR, median (IQR)	10.0	5.0–14.0	10.5	4.0–22.5	0.639
Trombocytes (×10^9^/L), median (IQR)	232.0	210–290	243.0	228–268	0.718
Fasting glucose (mmol/L), median (IQR)	5.3	4.8–5.4	5.4	4.7–6.0	0.652
Uric acid (µmol/l), median (IQR)	239	208–352	306	269–365	0.021*
CRP (mg/L), median (IQR)	2.5	1.0–4.5	3.8	2.1–10.0	0.103
Total cholesterol (mmol/L), median (IQR)	5.6	4.4–6.2	5.7	5.2–6.5	0.332
Triglycerides (mmol/L), median (IQR)	1.1	0.9–1.7	1.3	0.9–2.4	0.282
HLD (mmol/L), median (IQR)	1.3	1.2–1.7	1.3	1.1–1.7	0.482
LDL (mmol/L), median (IQR), median (IQR)	3.7	2.9–4.2	3.8	3.2–4.1	0.726
LVIDd (mm), median (IQR)	4.9	4.5–5.1	5.0	4.6–5.4	0.141
LA Diam (mm), median (IQR)	3.5	3.2–4.0	3.9	3.4–4.1	0.158
TAPSE (cm), median (IQR)	2.6	2.5–3.0	2.5	2.1–2.6	0.016*
MV E/A Ratio, median (IQR)	1.2	1.0–1.4	1.2	1.0–1.4	0.823
E/E Avg, median (IQR)	6.8	5.9–8.4	6.9	5.7–7.9	0.571
LVEF_BiP (%), median (IQR)	66.0	59.0–69.0	56.0	52.0–60.0	0.001*
BLC (pg/ml), median (IQR)	76.3	60.4–95.1	81.5	47.9–118.1	1.000
MIG (pg/ml) , median (IQR)	7.2	4.7–14.7	7.0	2.7–11.0	0.399
IL-17 (pg/ml), median (IQR)	0.29	0.15–0.43	.46	0.26–0.65	0.056
TNF L (pg/ml), median (IQR)	20.7	13.0–43.3	21.83	16.5–31.6	0.964
Adiponektin (ng/L), median (IQR)	8.6	6.0–13.6	5.9	4.7–7.8	0.036*
Leptin (pg/L), median (IQR)	11.8	8.9–23.8	14.1	8.8–19.5	0.682
Resistin (pg/ml), median (IQ R)	10.1	8.5–11.3	9.7	6.8–12.1	0.937

*BMI* body mass index, *PsA* psoriatic arthritis, *BP* blood pressure, *PASI* Psoriasis Area and Severity Index, *DAPSA* Disease Activity in Psoriatic arthritis, *ESR* Erythrocyte sedimentation rate, *CRP* C reactive proteine, *HDL* high-densitiy lipoprotein, *LDL* low density lipoprotein, *LVIDd* left ventricular internal diameter at end-diastole, *LA* left atrium, *TAPSE* tricuspid annular plane systolic excursion, *MV* mitral valve, *E/E* early diastolic mitral inflow velocity to early diastolic mitral annulus velocity, *GLS* global longitudinal strain, *LVEF BiP* left ventricular ejection fraction biplane, *BLC* B lymphocyte chemoattractant, *MIG* monokine induced by intereferon gamma, *IL* interleukin, *TNF L* tumor necrosis factor alfa, *IQR* interquartile range.

* statistically significant (P < 0.05).

Since there was a statistically significant difference in TAPSE between patients and controls, and also between patients with low, and moderate and high disease activity, we explored this association. When the study population was divided based on TAPSE ≤ 2.8 and > 2.8, we did not find significant differences in cytokine and adipokine levels between patients with mild PsA and controls. Moreover, no differences in inflammatory biomarkers were found in patients with low, and moderate to high disease activity when divided based on TAPSE ≤ 2.6 and > 2.6.

### Univariate and multivariate analysis

The main purpose of multivariate analysis was to assess two main features: a) the association between disease activity and GLS, IL-17 and BLC; b) myocardial dysfunction measured by GLS and IL-17 and adiponectin. However, we have firstly conducted univariate linear regression analysis which comprised of all variables that showed statistically significant differences or trend towards statistical significance in previous analyses.

In the first univariate analysis where we used logarithmically transformed DAPSA score as a dependent variable, age, PSA duration, serum CRP, triglycerides, IL-17 levels and GLS along with other echocardiographic parameters were associated with DAPSA (Table 4). Interestingly, BMI and serum BLC did not show significant correlation with DAPSA. Afterwards, we have conducted a multivariate analysis comprised of all parameters that showed significant association in aforementioned analysis, after which only CRP and triglyceride levels remained statistically significant (Model 2, Table 4). Due to limitations of univariate analysis discussed in statistical methods section, we have performed additional two different multivariate models which comprised of parameters that showed statistical significance in previous analyses. Hence, in model 3, disease activity expressed by DAPSA, was independently associated with serum IL-17 and GLS. This association remained statistically significant after adjustment for age and BMI in model 4 (Table 4).

**Table 4:** Univariate analysis and different multivariate analysis models in which logarithmically transformed DAPSA score was used as a dependent variable.

	B	SE	Beta	t	*P*
Model 1 (Univariate analysis)					
Log age	2.595	0.652	0.409	3.979	0.000
Log BMI	2.395	1.360	0.196	1.762	0.082
Log PSA duration	0.416	0.142	0.380	2.931	0.005
Log uric acid	0.264	0.487	0.080	0.543	0.590
Log CRP	0.549	0.105	0.604	5.253	0.000
Log triglycerides	0.596	0.228	0.359	2.613	0.012
Log BLC	0.103	0.311	0.044	0.331	0.742
Log MIG	0.334	0.180	0.239	1.856	0.069
Log IL17	0.561	0.102	0.551	5.486	0.000
Log adiponectin	0.064	0.369	0.021	0.173	0.864
Log Leptin	0.283	0.269	0.126	1.052	0.296
Log rezistin	−1.060	0.590	−0.211	−1.797	0.077
Log GLS	−5.920	1.011	−0.550	−5.854	0.000
Log LVIDd	4.033	1.988	0.225	2.029	0.046
Log LADiam	3.251	1.227	0.291	2.649	0.010
Log TAPSE	−5.436	0.824	−0.614	−6.600	0.000
Log MV	−1.175	0.436	−0.299	−2.695	0.009
Log EE	−0.189	0.811	−0.027	−0.233	0.816
Model 2 (Independent variables: Log age, Log CRP, Log triglycerides, Log IL-17, Log GLS)					
Log CRP	0.362	.109	0.478	3.308	0.002
Log triglycerides	0.486	.218	0.318	2.226	0.033
Model 3 (Independent variables: Log GLS, Log IL-17, Log BLC)					
Log IL-17	0.398	0.112	0.397	3.542	0.001
Log GLS	−4.365	1.305	−0.375	−3.346	0.001
Model 4 (Independent variables: Log GLS, Log IL-17, Log BLC, Log BMI and Log age)					
Log IL-17	0.415	0.105	0.418	3.963	< 0.001
Log GLS	−3.210	1.264	−0.277	−2.539	0.014
Age	0.045	0.015	0.303	3.065	0.003

*B* unstandardized coefficient; *SE* standard error; *Beta* Standard coefficient, *DAPSA* Disease Activity in Psoriatic arthritis, *PSA* psoriatic arthritis, *GLS* global longitudinal strain, *IL-17* interleukin 17, *CRP* C reactive protein, *BMI* body mass indeks, *LVIDd* left ventricular internal diameter at end-diastole, *LADiam* left atrium diameter, *TAPSE* tricuspid annular plane systolic excursion, *MV* mitral valve, *E/E* early diastolic mitral inflow velocity to early diastolic mitral annulus velocity, *GLS* global longitudinal strain, *BLC* B lymphocyte chemoattractant, *MIG* monokine induced by intereferon gamma.

Model 1—Univariate analysis (LogDAPSA set as dependent variable, independent variables were comprised of all cytokines, echo parameters and other parameters with statistical significant difference in prior analyses).

Model 2—Regional multivariate analysis where LogDAPSA was used as dependent variable, while independent variables were comprised of all parameters that showed statistical significant association in univariate analysis (Log Age, Log CRP, Log triglycerides, Log IL-17, Log GLS).

Model 3—Regional multivariate analysis where LogDAPSA was used as dependent variable, while independent variables were chosen at author’s discretion based on previous analyses (Log GLS, Log IL-17, Log BLC).

Model 4—Regional multivariate analysis where LogDAPSA was used as dependent variable, while independent variables were chosen at author’s discretion based on previous analyses (Log GLS, Log IL-17, Log BLC, BMI and age).

Second univariate analysis was carried out with logarithmically transformed GLS as a dependent variable (Table 5). Overall, the results of linear regression analysis corresponded well with previous analyses with few differences. In regression analysis the association between IL-17 and GLS became statistically significant, while the significance between adiponectin and GLS diminished (Model 1). In model 2 all aforementioned associations diminished, except for DAPSA score. In model 3, GLS was independently associated with only serum IL-17. However, after adjustment for age and BMI in model 4, the association between GLS and serum adiponectin emerged as statistically significant.

**Table 5:** Univariate analysis and different multivariate analysis models in which logarithmically transformed GLS was used as a dependent variable.

	B	SE	Beta	t	*P*
Model 1 (Univariate analysis)					
Log age	−0.155	0.064	−0.262	−2.417	0.018
Log BMI	−0.341	0.124	−0.298	−2.762	0.007
Log DAPSA	−0.051	0.009	−0.550	−5.854	0.000
Log Uric	−0.213	0.078	−0.375	−2.742	0.009
Log IL17	−0.037	0.011	−0.393	−3.549	0.001
Log adiponectin	0.044	0.034	0.155	1.300	0.198
Log TAPSE	0.431	0.082	0.528	5.276	0.000
Log LVEF	0.442	0.113	0.402	3.899	0.000
Model 2 (Independent variables: Log age, Log BMI, Log DAPSA, Log uric, Log IL-17, Log adiponectin)					
Log DAPSA	−0.048	0.010	−0.506	−4.972	0.000
Model 3 (Independent variables: Log IL-17 and Log adiponectin					
Log IL-17	−0.039	0.010	−0.407	−3.714	< 0.001
Log adiponectin	0.053	0.031	0.185	1.693	0.095
Model 4 (Independent variables: Log IL-17, Log adiponectin, Log age and Log BMI)					
Log IL-17	−0.035	0.010	−0.370	−3.468	0.001
Log adiponectin	0.067	0.031	0.233	2.195	0.032
Log age	−0.004	0.001	−0.274	−2.554	0.013

*B* unstandardized coefficient; *SE* standard error; *Beta* Standard coefficient, *DAPSA* Disease Activity in Psoriatic arthritis, *GLS* global longitudinal strain, *IL-17* interleukin 17, *BMI* body mass indeks, *TAPSE* tricuspid annular plane systolic excursion, *LVEF* left ventricular ejection fraction.

Model 1—Univariate analysis (LogGLS set as dependent variable, independent variables were comprised of all cytokines, echo parameters and other parameters with statistical significant difference in prior analyses).

Model 2—Regional multivariate analysis where LogGLS was used as dependent variable, while independent variables were comprised of all parameters that showed statistical significant association in univariate analysis (Log age, Log BMI, Log DAPSA, Log uric, Log IL-17, Log adiponectin).

Model 3—Regional multivariate analysis where LogGLS was used as dependent variable, while independent variables were chosen at author’s discretion based on previous analyses (*Log IL-17 and Log adiponectin*).

Model 4—Regional multivariate analysis where LogGLS was used as dependent variable, while independent variables were chosen at author’s discretion based on previous analyses (Log IL-17, Log adiponectin, Log age and Log BMI).

## Discussion

This study found an association between psoriatic arthritis disease activity and degree of subclinical myocardial dysfunction. We found that elevated IL-17 and decreased adiponectin may be associated with subclinical myocardial dysfunction in patients with PsA. This is the first study to examine the association between adipokines, proinflammatory cytokines, and subclinical myocardial dysfunction in patients with PsA.

Patients with psoriatic arthritis have increased cardiovascular risk and mortality when compared to healthy controls^2–4^. Underlying chronic inflammation and elevated circulating proinflammatory mediators have been implicated, leading to endothelial dysfunction and accelerated atherosclerosis^7,26,27^. A recent metanalysis showed a significantly increased incidence of myocardial infarction and heart failure in patients with PsA in comparison to healthy controls^2^.

Several studies have shown early myocardial involvement and more frequent subclinical left ventricular dysfunction in patients with PsA^9–13^. STE is a novel, more sensitive technique used to detect impaired LV function, which evaluates myocardial deformation in multidimensional planes by measuring global longitudinal strain (GLS), circumferential strain, radial strain, and apical rotation^28^. Compared to conventional echocardiography and LVEF (Simpson biplane), GLS has superior prognostic value for predicting major adverse cardiac events and all-cause mortality^29^.

Our study confirmed a higher prevalence of subclinical myocardial dysfunction, depicted by GLS, in patients with PsA in comparison to controls, and a significant negative correlation between disease activity and myocardial function, depicted by GLS and LVEF. Yilmazer et al., also reported reduced GLS, circumferential strain, and radial strain in 31 patients with PsA and without traditional CV risk factors in comparison to 20 sex and age matched controls; however, no correlation between disease activity and myocardial deformation was found^9^. In accordance with our results, Lo Gullo et al. reported impaired GLS in 35 patients with PsA without traditional CV risk factors when compared to healthy controls, and a positive correlation between disease activity, assessed by Disease activity score-28 and myocardial strain impairment^11^.

We explored possible mechanisms of early myocardial dysfunction in patients with PsA, with an emphasis on the role of adipokines. Patients with PsA have increased BMI compared to the general population^30^, and evidence suggests that obesity increases the risk of PsA and is a predictor of poor treatment response^31,32^. Adipokines are metabolically active cytokines that are released from visceral fat adipocytes, which have anti-inflammatory and cardioprotective effects (adiponectin, omelin, apelin), as well as proinflammatory effects (resistin, leptin, visfatin, TNF alpha)^20^. We found that PsA patients with decreased GLS had lower adiponectin levels.

Adiponectin has anti-inflammatory and antiatherogenic effects and improves insulin resistance^20^. A negative correlation between adiponectin and central obesity is observed in adults, with lower adiponectin levels associated with metabolic syndrome, generalized atherosclerosis, coronary artery disease, and acute coronary syndromes^33,34^. However, increased adiponectin levels are seen in patients with heart failure and left ventricular systolic dysfunction, and are positively correlated with the severity of heart failure and mortality^35,36^. This “adiponectin paradox’’ is attributed to adiponectin resistance in advanced stages of CVD, and compensatory adiponectin increase, which highlights the complexity of adiponectin signaling. In our study, lower adiponectin levels were found in patients with more impaired myocardial function (lower GLS), and this subgroup had increased BMI. In most studies, hypoadiponectinemia was found in patients with obesity, metabolic syndrome and diabetes mellitus^33,34^, which could partly explain these results. However, patients with diabetes and components of the metabolic syndrome were excluded from our study. Furthermore, the average BMI in the subgroup of patients with lower GLS was 26.3 kg/m^2^, which is in the overweight, and not obese range. Therefore, hypoadiponectinemia may be negatively associated with myocardial function, irrespective of BMI, which was confirmed by multivariate analysis.

We also examined the role of other adipokines (resistin, leptin, TNF alfa) on PsA disease activity, and their associations with subclinical myocardial dysfunction, but we did not find any statistically significant associations. Furthermore, no differences in these variables were found in patients with PsA versus controls. These results are not in agreement with previous studies^17,20,37^, that for the most part, showed increased proinflammatory cytokines in patients with PsA. These conflicting results can be attributed to the exclusion of patients with metabolic disorders, CV disease, and traditional cardiovascular risk factors from our study.

The association between proinflammatory cytokines and subclinical myocardial dysfunction was also assessed in patients with PsA. We found that patients with a greater degree of myocardial dysfunction had elevated IL-17A levels. As mentioned earlier, disease activity measured by DAPSA, was correlated with the degree of myocardial dysfunction. IL-17 is one of the main proinflammatory cytokines that potentiates the local inflammation of PsA-affected joints and entheses. Some studies suggest that the increased CV risk in patients with psoriasis, could be attributed to the inflammatory disease burden, mediated by IL-17^38^. IL-17 promotes endothelial dysfunction, and elevated IL-17 has also been observed in patients with acute coronary artery syndromes in comparison to patients with stable coronary artery disease^39^. However, the role of IL-17 on subclinical myocardial dysfunction in patients with inflammatory arthritis, including PsA, has not been investigated thus far.

A positive correlation between IL-17A and DAPSA score was found in this study. Also, GLS was independently associated with serum IL-17. This suggests that IL-17 may be associated with the development of subclinical myocardial dysfunction in patients with higher PsA disease activity. Evidence suggests that IL-17 produced from visceral fat tissue, could mediate the development of systemic atherosclerosis^40^, which could also explain the potential association with myocardial dysfunction. However, the association between IL-17 and subclinical myocardial dysfunction remained after accounting for BMI and age as confounding factors, which strengthens this conclusion.

### Study limitations

Small sample size is the main study limitation. The cross-sectional design of study limits the assessment of proinflammatory cytokines and adipokines over the time. The results of multivariate analysis should be interpreted with caution, because of the small sample size. This is a proof-of-concept study, and lays a solid foundation for planning and conducting future studies in this field.

## Conclusion

Our study confirmed that patients with PsA without traditional CV risk factors and higher disease activity, have an increased prevalence of subclinical myocardial dysfunction. Decreased adiponectin and increased IL-17 levels may be associated with impaired myocardial function in these patients, but future studies on larger samples are need to confirm these causal relationships. If future studies confirm these causal relationships, inflammatory biomarkers could be used to identify high-risk patients and implement preventative cardiovascular measures in order to decrease cardiovascular morbidity and mortality in these patients.

## Supplementary Information

Supplementary Information.

## Data Availability

All data generated or analysed during this study are included in this published article and its supplementary information files.
